# One-Step Synthesis of Highly Efficient Oligo(phenylphosphonic Dihydroxypropyl Silicone Oil) Flame Retardant for Polycarbonate

**DOI:** 10.3390/polym11121977

**Published:** 2019-12-01

**Authors:** Yihui Qiao, Yanbin Wang, Menghao Zou, Dehuan Xu, Yingtong Pan, Zhonglin Luo, Biaobing Wang

**Affiliations:** Jiangsu Key Laboratory of Environmentally Friendly Polymeric Materials, School of Materials Science and Engineering, Jiangsu Collaborative Innovation Center of Photovolatic Science and Engineering, Changzhou University, Changzhou 213164, Jiangsu, China; qiaoyihui666@163.com (Y.Q.); 15861172153@163.com (M.Z.); xdh1103793517@163.com (D.X.); 13616158671@163.com (Y.P.); zhonglinluo@cczu.edu.cn (Z.L.)

**Keywords:** flame retardant, polycarbonate, combustion behavior, synergistic effect

## Abstract

A highly efficient flame retardant and smoke suppression oligomer, oligo(phenylphosphonic dihydroxypropyl silicone oil) (PPSO), was synthesized by a one-step reaction. The chemical structure of PPSO was confirmed by Fourier transform infrared (FTIR), ^31^P nuclear magnetic resonance (^31^P NMR), and ^29^Si nuclear magnetic resonance (^29^Si NMR). The flame-retardant effect of PPSO on the polycarbonate (PC) matrix was investigated by limiting oxygen index, UL-94 vertical burning test, and cone calorimetry, respectively. The results showed that PC/PPSO composites passed UL-94 V-0 rate testing with only 1.3 wt. % PPSO. Furthermore, the incorporation of PPSO can suppress the release of smoke. The flame-retardant mechanism was also investigated via thermogravimetric analysis-fourier transform infrared spectroscopy (TG-FTIR), field-emission scanning electronic microscopy (FE-SEM), X-ray photoelectron spectroscopy (XPS) and Raman spectroscopy. From the result of pyrolysis gas and char residue, PPSO played a synergistic flame-retardant mechanism including the gas phase and the condensed phase.

## 1. Introduction

Polycarbonate (PC) has become the fastest growing general engineering plastic among the five engineering plastics due to its good physical and chemical properties, including excellent transparency, high mechanical strength, good thermal stability, and high heat distortion temperature [1,2,3]. Moreover, PC exhibits more superior flame retardancy than ordinary plastics owing to its relatively high limiting oxygen index (22%–25%) and flame-retardant grade (UL-94 V-2) [4,5]. Nevertheless, it is still difficult to meet the harsh requirement of flame retardancy in some specific applications. Furthermore, melt dripping occurs during PC combustion, which may cause a communicated fire [6,7,8]. Therefore, it is necessary to improve the flame retardancy of the PC matrix.

Until now, many kinds of flame retardants containing halogen, sulfonate, phosphorus, and other elements have been designed for PC [9,10,11,12]. Among these flame retardants, phosphorus- and silicon-containing compounds as halogen-free flame retardants have attracted much attention due to their high efficiency, smoke suppression performance, and low toxicity [13,14,15,16,17,18]. More importantly, phosphorus- and silicon-containing flame retardants could enrich the PC surface during combustion, which is useful for char formation and improving the quality of the carbon layer [19,20]. For example, Liu et al. designed a novel flame retardant named PSiN, containing silicon and nitrogen, and it was used together with 9,10-dihydro-9-oxa-10-phosphaphenanthrene-10-oxide (DOPO) to fabricate a flame-retardant system for epoxy resins (EP) [20]. When 3% PSiN and 7% DOPO were incorporated, the LOI value of EP was found to be 34% and the UL-94 test was found to be class V-0. The microstructures observed by scanning electron microscopy and FTIR indicated that the surface of the char for the EP/DOPO/PSiN system holds a more cohesive and dense char structure when compared to the pure EP and EP/DOPO systems. Many works have been performed on the compounds containing two or more flame-retardant elements including phosphorus, nitrogen, sulfur, and boron [21,22,23,24,25]. However, few studies have reported on flame retardants containing both phosphorus and silicon, and the weight ratios of the flame retardants were too high [26,27]. For example, Yang group designed a novel polyhedral oligomeric silsesquioxane containing 9,10-dihydro-9-oxa-10-phosphaphenanthrene-10-oxide (DOPO-POSS) and incorporated it into the PC matrix, and the PC/DOPO-POSS composites passed UL-94 V-0 rate testing, but the weight ratio (6 wt. %) of the flame retardant was relative high [26]. Recently, a polymer flame retardant named HSPCTP has been prepared by our group and incorporated into the PC matrix, and the PC/HSPCTP composites passed UL-94 V-0 rating testing with only 3 wt. % HSPCTP [13]. This excellent property resulted from the synergistic effect of a gas phase flame retardant and condensed phase flame. These results suggest that phosphorus and silicon are a good combination to prepare a flame retardant. But the synthesis of this flame retardant is complicated. Therefore, it is worth preparing a high efficient flame retardant with a simple method.

In this study, a novel oligomer flame retardant containing phosphorus and silicon was synthesized by a one-step reaction and then blended with PC. Combining the advantages of phosphorus and silicon, the PC/oligo(phenylphosphonic dihydroxypropyl silicone oil) (PPSO) composites passed UL-94 V-0 rating testing with just only 1.3 wt. % PPSO. Furthermore, the flame-retardant mechanism was also investigated via TG-IR, FE-SEM, XPS and Raman spectroscopy.

## 2. Materials and Methods 

### 2.1. Materials

Dihydroxypropyl silicone oil (DHSO, 40–50 mg KOH/g of hydroxyl value) was provided by Hong sheng Chemical Products Co., Ltd. (Guangzhou, China), and then dehydrated by magnesium sulfate anhydrous. Phenylphosphonic dichloride (PPDC) was purchased from Alpha Chemical Co., Ltd. (Zhengzhou, China). Tetrahydrofuran (THF), triethylamine (TEA), and dichloromethane (DCM) were purchased from Sinopharm Chemical Reagent Co., Ltd. (Shanghai, China). TEA was stored with a 4A molecular sieve and THF was dried over sodium before using. Talcum powder (Talc) was purchased from Quanzhou Xufeng Powder Raw Material Co., Ltd. (Quanzhou, China). Polycarbonate (PC) was supplied by Korea LG Co., Ltd. (Seoul, Korea). PC and Talc were dried in a vacuum oven at 110 °C for 12 h before mix blending.

### 2.2. Synthesis of Oligo(Phenylphosphonic Dihydroxypropyl Silicone Oil)

**Scheme 1:**
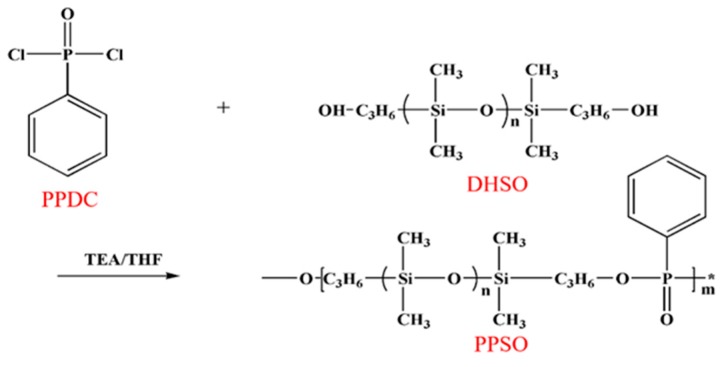
The synthetic route of oligo(phenylphosphonic dihydroxypropyl silicone oil) (PPSO).

The synthetic route of oligo(phenylphosphonic dihydroxypropyl silicone oil) (PPSO) is depicted in Scheme 1, and the experimental process is described as follows: 

Dihydroxypropyl silicone oil (18.03 g, 10 mmol), TEA (2.46 g, 24 mmol), and THF (20 mL) were added into a 250 mL four-necked flask. The mixture was cooled to 0 °C in an ice/water bath, and then the solution of phenylphosphonic dichloride (2.34 g, 12 mmol) in THF (30 mL) was added dropwise into the flask. The reaction mixture was stirred at 0 °C for 2 h and heated slowly to 70 °C for 12 h under nitrogen atmosphere. The triethylamine salt was removed by vacuum filtration under reduced pressure, and THF was removed by rotary evaporation. Next, the crude product dissolved in dichloromethane was washed with water several times. Finally, the product was dried at 100 °C for 12 h, and a colorless oily liquid was obtained, with a yield of 95% (*M*_n_ = 1300, *M*_w_ = 3571).

### 2.3. Preparation of Flame-Retardant PC Blends

A series of PC-based blends with various PPSO concentration were prepared by the melt blending method on an internal mixer at 240 °C for 5 min. Then the mixture was extracted, cooled to room temperature, and cut into granules.

### 2.4. Characterization

The Fourier transform infrared (FTIR) spectra were investigated by a PerkinElmer instrument (Waltham, Massachusetts, USA) at room temperature. The samples were mixed with KBr pellets and scanned 32 times over a spectral range of 4000–400 cm^−1^ with a resolution of 4 cm^−1^.

^29^Si NMR and ^31^P NMR spectra were conducted on a Bruker AV II- 400 MHz (Brook, Austria) at room temperature using CDCl_3_ as the solvent.

Gel-permeation chromatography (GPC) was performed on a Waters Breeze GPC (Massachusetts, USA). The solvent was tetrahydrofuran and the flow rate was 1.0 mL/min.

The limiting oxygen indexes were measured by an LOI analyzer (JF-3, Jiang Ning Co. Ltd., Nanjing, China) with sheet dimensions of 130 mm × 6.5 mm × 3 mm according to GB/T 2406-93 standard.

The vertical burning test (UL-94) of each sample was evaluated on a CZF-5-type instrument (Shine Ray Instrument Co. Ltd., Nanjing, China) with sheet dimensions of 130 mm × 13 mm × 2 mm according to ASTM D3801. An average of at least five replicas were used for each sample.

Thermogravimetric analysis (TGA) was conducted on a PerkinElmer TGA 4000 (Waltham, Massachusetts, USA) with a heating rate of 10 °C/min under nitrogen atmosphere and air atmosphere at temperatures ranging from 30 to 800 °C. TGA was coupled with Fourier transform infrared spectroscopy (TGA-FTIR), and the measurements were carried out under nitrogen atmosphere at a heating rate of 5 °C/min from 30 to 800 °C.

The fire behavior of the PC composites was evaluated via a cone calorimeter device (Fire Testing Technology, East Grin stead, UK) according to ISO 5660-1. The square sample with the dimension of 100 mm × 100 mm × 3 mm was exposed to a radiant cone at a heat flux of 35 kW/m^2^.

The surface of char residue after cone calorimetry tests and fractured surface (in liquid nitrogen) of the PC composites were investigated by field-emission scanning electronic microscopy (FE-SEM) Zeiss SUPRA55 (Jena, Germany). The surface of samples was sputter-coated with a conductive gold layer before observation.

Raman spectroscopy measurement was carried out with a DXR laser Raman spectrometer (Thermo scientific, Massachusetts, USA) using a 532 nm helium-neon laser line at room temperature.

X-ray photoelectron spectroscopy (XPS) was determined by an ESCALAB 250XI system (ThermoFischer, Massachusetts, USA).

## 3. Results and Discussion

### 3.1. Structural Characterization of PPSO

**Figure 1:**
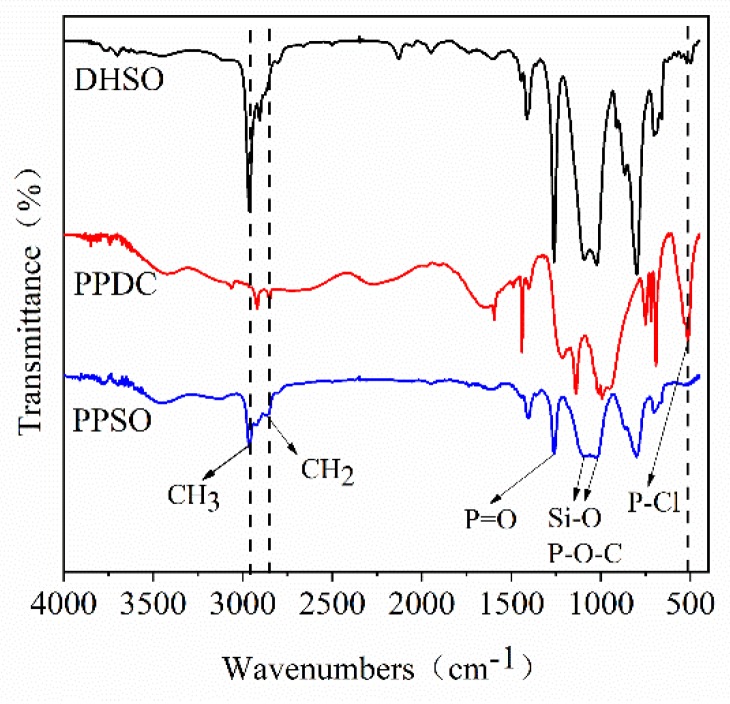
FTIR spectra of dihydroxypropyl silicone oil (DHSO), phenylphosphonic dichloride (PPDC), and PPSO.

**Figure 2:**
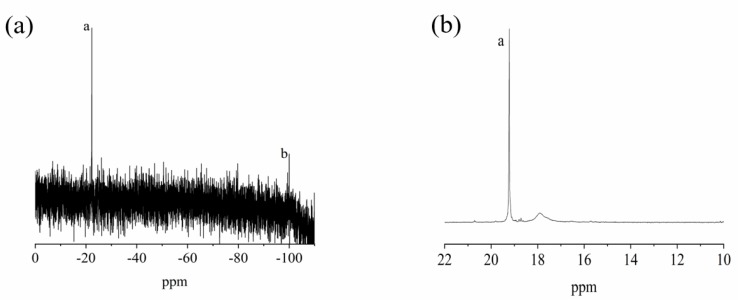
^29^Si NMR (**a**) and ^31^P NMR (**b**) spectra of PPSO.

The chemical structure of PPSO was confirmed by FTIR, ^29^Si NMR, and ^31^P NMR, respectively. Figure 1 shows the FTIR spectra of PPDC, DHSO, and PPSO. The characteristic peak at 515 cm^−1^ attributed to the P–Cl group of PPDC disappeared in the FTIR spectra of PPSO, which suggests that the chlorine is almost replaced in the final product. For the PPSO, the broad peak around 1020 cm^−1^ is assigned to the Si–O–Si group and P–O–C bond, and the peaks at 2900 and 2800 cm^−1^ are attributed to the presence of –CH_3_ and –CH_2_ group. The peak located at 1250 cm^−1^ is attributed to the P=O bond. The existence of a P–O–C bond also indicates that the PPSO was successfully synthesized. As shown in Figure 2a, there are two apparent peaks at −22.30 and −99.85 ppm, which means two major different silicon environments (Si–O and Si–C) in the PPSO. As shown in the Figure 2b, the main peak located at 19.22 ppm is assigned to the phosphorus connecting to DHSO. In summary, the oligomer flame retardant (PPSO) was successfully synthesized.

### 3.2. Flame Retardancy

The flame retardancy of the pure PC and PC/PPSO composites were measured via LOI and UL-94 testing, and the experimental data are listed in Table 1. The pure PC and PC/PPSO-0 samples exhibited a UL-94 V-2 rate due to serious melt dripping. The melt dripping phenomenon was suppressed obviously through adding a low loading level (1 wt. %) of PPSO. Furthermore, the incorporation of 1.3 wt. % PPSO gave the PC composites UL-94 V-0 rating with 29% of LOI.

### 3.3. Thermal Degradation

**Figure 3:**
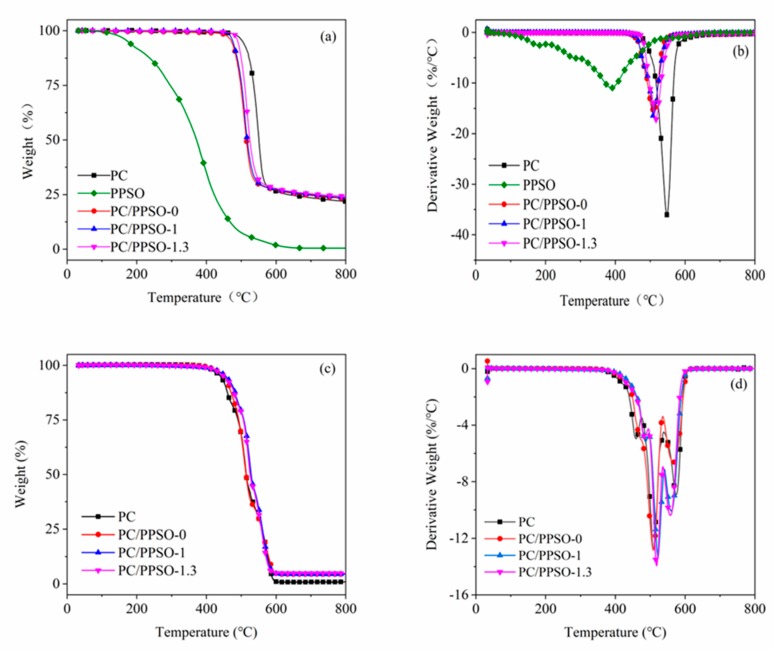
TGA (**a**,**c**) and derivative thermogravimetry (DTG) (**b**,**d**) curves of PC/PPSO composites in nitrogen (**a**,**b**) and air (**c**,**d**) atmospheres.

Thermogravimetric analysis (TGA) and derivative thermogravimetry (DTG) were used to investigate the thermal degradation behavior of the pure PC matrix and PC/PPSO composites under both nitrogen and air atmospheres, and their data are summarized in Table 2. The initial decomposition temperature (*T*_5%_) is defined as the temperature at 5 wt. % mass loss. *T*_max_ is the temperature corresponding to the maximum decomposition rate. As shown in Figure 3a,b, the *T*_5%_ value of the PC/PPSO-0 sample is much lower than that of the pure PC matrix under nitrogen atmosphere, revealing that the incorporation of talc promotes the thermal decomposition of the PC matrix due to the existence of the hydroxyl group on the talc surface. On the other hand, by increasing the weight ratio of PPSO, the *T*_5%_ value increased. This is because the flame-retardant PPSO decomposed at a lower temperature and released the phosphorus-containing group, which can form a phosphorus-rich protective carbon layer, as discussed later. The silicon-containing group (Si–O or Si–C) improves thermal stability and inhibits further decomposition of the PC matrix, which is consistent with results reported in previous literature [19,20]. Furthermore, it is found that the char residue also increased from 23.8 wt. % for PC/PPSO-0 composites to 24.7 wt. % for PC/PPSO-1.3 composites, indicating that PPSO plays a remarkable carbonization effect. 

As shown in Figure 3c,d, the thermal oxidative decomposition process of the PC matrix and PC/PPSO composites is more complicated than that under nitrogen atmosphere, and they exhibited three decomposition stages. The first and second decomposition stages are mainly ascribed to the decomposition of PPSO and the main chain of the PC matrix. The third stage is the further oxidative decomposition of the carbon layer formed in the previous stages. Pure PC is almost completely burned in the air atmosphere, and the carbon residue rate of PC/PPSO composites increased gradually by increasing the weight ratio of PPSO.

### 3.4. Fire Behavior of PC-Based Blends

**Figure 4:**
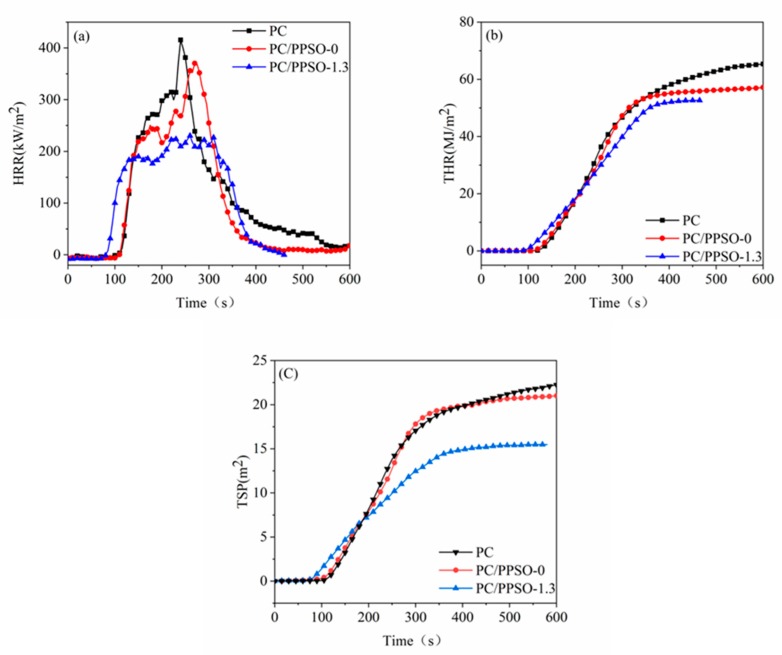
Heat release rate (HRR) (**a**), total heat release (THR) (**b**), and total smoke production (TSP) (**c**) curves of PC and its blends.

A cone calorimeter (CC) test was used to further investigate the fire behaviors of the pure PC matrix and its blends, which can provide multiple parameters such as time to ignition (TTI), peak heat release rate (PHRR), time to peak heat release rate (*t*_P_), total heat release (THR), total smoke production (TSP), and the average effective heat of combustion of volatiles (Av-EHC). Figure 4 illustrates the heat release rate (HRR), THR, and TSP curves of the pure PC matrix and its blends; their corresponding data are summarized in Table 3. Obviously, the PC/PPSO-1.3 sample gave a lower TTI value (60 s) than that of the pure PC matrix (96 s) and PC/PPSO-0 (93 s) samples, which is attributed to the early decomposition of PPSO to promote char formation. This is consistent with TGA results. The heat release rate (HRR) is considered to be the most important parameter for predicting the risk of combustion [28]. As shown in Figure 4a, the HRR value increased initially and then decreased for all specimens with increasing time. The difference is that the PC/PPSO-1.3 sample gave the lowest PHRR value (230 kw/m^2^) and the highest t_p_-HRR value (280 s) compared with the pure PC matrix and PC/PPSO-0 samples, which means that the early decomposition of the flame retardant is beneficial to the formation of effective carbon layers. Toxic smoke is widely known to be the most important factor endangering human life safety. Compared with the pure PC, PC/PPSO composites display much lower TSP values, revealing that PPSO shows a good smoke-suppressing effect on PC. The fire performance index (FPI) is defined as the ratio of TTI to PHRR, and a lower FPI value means a higher fire risk and worse fire resistance [29]. The FPI values of the pure PC, PC/PPSO-0, and PC/PPSO-1.3 are calculated to be 0.23, 0.25, and 0.26, respectively, which suggest that PPSO exhibited an obvious flame-retardant effect.

### 3.5. Flame Retardant Mechanism

**Figure 5:**
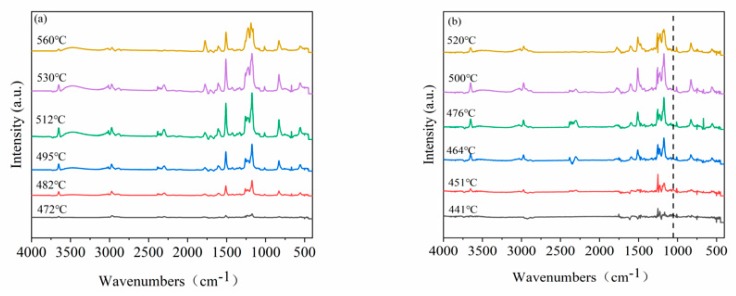
TG-FTIR spectra of PC (**a**) and PC/PPSO-1.3 (**b**) at different decomposition temperatures.

TG-FTIR is usually adopted to analyze the gaseous products during the thermal degradation process. For the pure PC sample as shown in Figure 5a, there are some characteristics observed as follows: H_2_O (3650 cm^−1^), CH_4_ (2950 cm^−1^), CO_2_ (2200 cm^−1^), phenol derivatives (Caromatic-H, 1500 cm^−1^), aromatic ethers (1200 cm^−1^), hydrocarbons (C–H, 1172 cm^−1^), etc. [4]. For the PC composite with 1.3 wt. % of PPSO, in addition to these above-mentioned peaks, a new characteristic absorption peak at 1020 cm^−1^ was observed as shown in Figure 5b, which is attributed to the P–O–C group. This result provides direct evidence that the PC/PPSO composites released some phosphorus gases (PO•, PO2• etc.) [30] during the combustion process; in other words, the flame-retardant PPSO played a gas phase flame-retardant mechanism.

**Figure 6:**
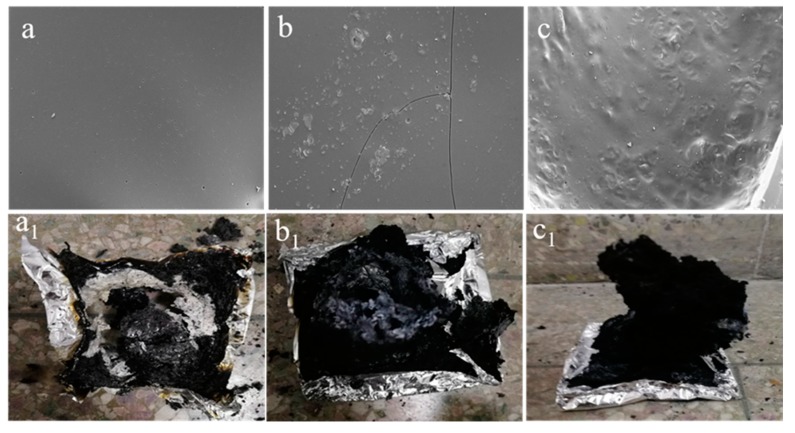
SEM images (1000×) and digital photos of char residue for pure PC (**a**,**a_1_**), PC/PPSO-0 (**b**,**b_1_**), and PC/PPSO-1.3 (**c**,**c_1_**) obtained from cone calorimeter test.

To further investigate the quality of the residual char, the residues after the cone calorimeter test were observed by SEM. As shown in Figure 6, the char residue of pure PC is incomplete and damaged. With the addition of talc and PPSO, char residues became dense and continuous. In more detail, for the pure PC sample, a thin and brittle carbon layer was observed in Figure 6a during the combustion process, and the surface of pure PC residue displays many holes with different sizes. This is because some of the flammable volatiles formed during the thermal degradation can penetrate the carbon layer into the flame zone. For the FR-0 sample, the surface of the carbon layer shows few holes and some cracks as shown in Figure 6b, which suggest that the carbon layer is formed readily with the incorporation of the talc, but it is still not strong enough to insulate the heat and flammable volatiles. For the PC/PPSO composite with 1.3 wt. % PPSO, a much denser and expanded carbon layer was formed as shown in Figure 6c. The early decomposition of PPSO, as demonstrated by the TGA results, produces PO• free radicals which can capture the flammable free H• and OH• radicals generated during the combustion of the matrix, and a stable carbon layer is thus formed [31]. The carbon layer helps to block the transfer of heat and oxygen, but also prevents the matrix from further burning; in other words, the flame-retardant PPSO also acts as the effect of the condensed phase flame retardant.

**Figure 7:**
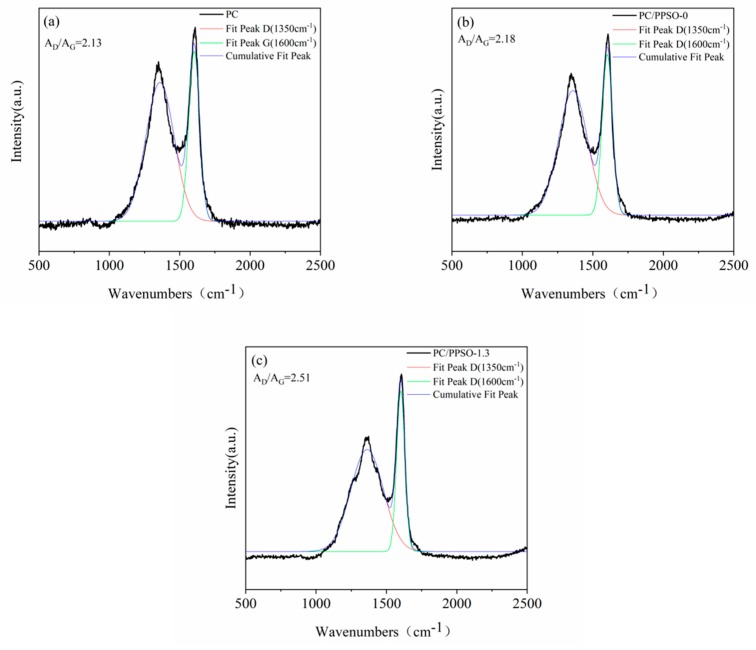
Raman spectra of char residue for pure PC (**a**), PC/PPSO-0 (**b**) and PC/PPSO-1.3 (**c**).

Raman spectroscopy is an effective method to investigate the quality of carbon layers, and the char residue spectra of pure PC, FR-0, and FR-1.3 are shown in Figure 7. There are two distinct bands for all samples: G bands (about 1600 cm^−1^) and D bands (about 1350 cm^−1^). The degree of graphitization of the carbon residue is determined by the peak area ratio (*A*_D_/*A*_G_) of the D and G bands, and the higher *A*_D_/*A*_G_ value indicates better carbon structure and higher thermal stability [32,33]. The *A*_D_/*A*_G_ values of the char residue for the pure PC, FR-0, and FR-1.3 are calculated to be 2.13, 2.18, 2.51, respectively, revealing that the FR-1.3 sample has the highest degree of graphitization, which means the transfer of oxygen and heat are restricted. This is consistent with the improved flame retardancy with the incorporation of PPSO as discussed previously.

**Figure 8:**
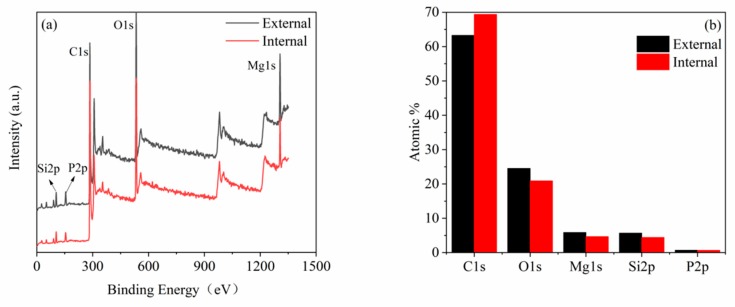
XPS spectra for external and internal char residue of the PC/PPSO-1.3 sample.

XPS test was conducted to analyze the elemental composition of the carbon layer, and the calculated elemental composition is summarized in Table 4. As shown in Figure 8, although the external and internal char residues have the same elements, it is worth noting that the Si element is richer in the external carbon layer than in the internal carbon layer. This is probable because PPSO tends to migrate to the polymer surface during the combustion process, resulting in a protective layer rich in Si–C and Si–O. It also indicates that silicon fragments play flame-retardant roles in the condensed phase.

**Figure 9:**
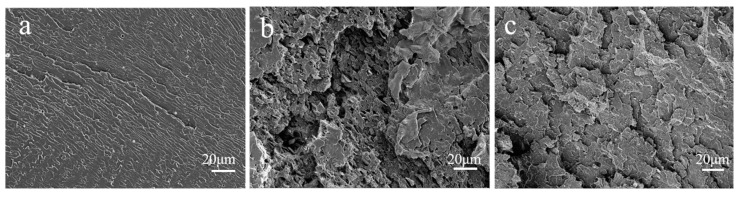
Fracture surface morphology of pure PC (**a**), PC/PPSO-0 (**b**), and PC/PPSO-1.3 (**c**).

The fracture surface morphology of the PC matrix and PC/PPSO composites was investigated by FE-SEM as shown in Figure 9. The fracture surface of PC is smooth with the incorporation of talc into the PC matrix, and there are a lot of holes on the PC matrix surface, which became rough because talc has poor compatibility with the PC matrix. Encouragingly, the PC/PPSO composite with 1.3 wt. % of PPSO became smooth compared with PC/PPSO-0 composite, which indicates that flame-retardant PPSO can effectively improve the compatibility of talc and the PC matrix. This is probable because flame-retardant PPSO contains two parts, phenylphosphonic (the organic part) and hydroxypropyl silicone oil (the inorganic part), which possess similar structures to the PC matrix and talc, respectively.

## 4. Conclusions

A novel phosphosilicone-containing flame-retardant oligomer (PPSO) was successfully synthesized by nucleophilic substitution between phenylphosphonic dichloride and dihydroxypropyl silicone oil. The addition of PPSO can effectively improve the compatibility of talc in the PC matrix, and thus showed a highly efficient effect on the flame retardancy of the PC. The PC/PPSO composite passed the UL-94 V-0 rate with a low loading level of 1.3 wt. %. The cone calorimetry testing results showed that the addition of the PPSO suppressed the smoke release and reduced the fire risk obviously. The excellent flame-retardant effect of PPSO is ascribed to the synergistic interaction between the gas phase and the condensed phase.

## Figures and Tables

**Table 1:** Flame retardancy for polycarbonate (PC) and its blends.

Sample	PC (phr)	Talc (phr)	PPSO (phr)	LOI (%)	UL-94 Rating (2 mm)	Dripping or not
PC	100	0	0	25	V-2	Yes
PC/PPSO-0	100	5	0	26	V-2	Yes
PC/PPSO-1	100	5	1	28	V-1	No
PC/PPSO-1.3	100	5	1.3	29	V-0	No
PC/PPSO-1.6	100	5	1.6	30	V-0	No

**Table 2:** TGA data of the PC and PC/PPSO composites.

Sample	Nitrogen			Air				
	*T*_5%_ (°C)	*T*max (°C)	Residues at 750 °C (%)	*T*_5%_ (°C)	*T*max_1_ (°C)	*T*max_2_ (°C)	*T*max_3_ (°C)	Residues at 750 °C (%)
PC	504	549	22.6	440	459	509	575	0.8
PC/PPSO-0	472	510	23.8	451	467	510	567	4.4
PC/PPSO-1	476	509	24.2	455	487	522	561	4.5
PC/PPSO-1.3	490	517	24.7	449	483	519	558	4.9

**Table 3:** Cone calorimeter data for the PC and its blends.

Sample	PC	PC/PPSO-0	PC/PPSO-1.3
TTI (s)	96	93	60
PHRR (kw/m^2^)	416	370	230
t_p_-HRR (s)	220	270	280
THR (MJ/m^2^)	65.6	62.5	52.6
TSP (m^2^)	27.1	21.6	15.4
av-EHC (MJ/kg)	20.1	18.9	17.9
FPI	0.23	0.25	0.26

**Table 4:** XPS results of PC/PPSO-1.3 sample.

Sample	C1s (%)	O1s (%)	Mg1s (%)	Si2p (%)	P2p (%)
External	63.28	24.52	5.84	5.69	0.68
Internal	69.36	20.90	4.67	4.40	0.66

## References

[1] Wang T., Yan M., Sun X., Quan D. (2015). The mechanical and biological properties of polycarbonate-modified F127 hydrogels after incorporating active pendent double-bonds. Polymer.

[2] Arjun G.N., Ramesh P. (2012). Structural characterization, mechanical properties, and in vitro cytocompatibility evaluation of fibrous polycarbonate urethane membranes for biomedical applications. J. Biomed. Mater. Res..

[3] Levchik S.V., Weil E.D. (2005). Overview of recent developments in the flame retardancy of polycarbonates. Polym. Int..

[4] Jang B.N., Wilkie C.A. (2004). A TGA/FTIR and mass spectral study on the thermal degradation of bisphenol A polycarbonate. Polym. Degrad. Stab..

[5] Huang K., Yao Q. (2015). Rigid and steric hindering bisphosphate flame retardants for polycarbonate. Polym. Degrad. Stab..

[6] Feng J., Hao J., Du J., Yang R. (2010). Flame retardancy and thermal properties of solid bisphenol A bis(diphenyl phosphate) combined with montmorillonite in polycarbonate. Polym. Degrad. Stab..

[7] Zhao W., Li B., Xu M., Yang K., Lin L. (2013). Novel intumescent flame retardants: Synthesis and application in polycarbonate. Fire Mater..

[8] Weil E.D., Levchik S.V. (2008). Flame retardants in commercial use or development for polyolefins. J. Fire Sci..

[9] Hu Z., Chen L., Zhao B., Luo Y., Wang D.Y., Wang Y.Z. (2011). A novel efficient halogen-free flame retardant system for polycarbonate. Polym. Degrad. Stab..

[10] Bozi J., Czégény Z., Meszaros E., Blazso M. (2007). Thermal decomposition of flame retarded polycarbonates. J. Anal. Appl. Pyrolysis.

[11] Ishikawa T., Maki I., Koshizuka T., Ohkawa T., Takeda K. (2004). Effect of perfluoroalkane sulfonic acid on the flame retardancy of polycarbonate. J. Macromol. Sci. Part A.

[12] Feng J., Hao J., Du J. (2012). Some developments in halogen-free flame retardancy of polycarbonate and its blends. Fire and Polymers VI: New Advances in Flame Retardant Chemistry and Science.

[13] Jiang J.C., Wang Y.B., Luo Z.L. (2019). Design and application of highly efficient flame retardants for polycarbonate combining the advantages of cyclotriphosphazene and silicone oil. Polymers.

[14] Lu S.Y., Hamerton I. (2002). Recent developments in the chemistry of halogen-free flame retardant polymers. Prog. Polym. Sci..

[15] Ding H., Huang K., Li S., Xu L., Xia J., Li M. (2017). Flame retardancy and thermal degradation of halogen-free flame-retardant biobased polyurethane composites based on ammonium polyphosphate and aluminium hypophosphite. Polym. Test..

[16] Mauerer O. (2005). New reactive, halogen-free flame retardant system for epoxy resins. Polym. Degrad. Stab..

[17] Rault F., Giraud S., Salaün F., Almeras X. (2015). Development of a halogen free flame retardant masterbatch for polypropylene fibers. Polymers.

[18] Dang X.R., Bai X., Zhang Y. (2010). Thermal degradation behavior of low-halogen flame retardant PC/PPFBS/PDMS. J. Appl. Polym. Sci..

[19] Yang Y., Kong W., Cai X. (2016). Two phosphorous-containing flame retardant form a novel intumescent flame-retardant system with polycarbonate. Polym. Degrad. Stab..

[20] Liu C., Qiang Y. (2017). Design and synthesis of efficient phosphorus flame retardant for polycarbonate. Ind. Eng. Chem. Res..

[21] Liu S.M., Yang Y., Jiang Z.J., Zhou Y.H., Zuo J., Zhao J.Q. (2012). Synergistic flame retardant effect of poly(ether sulfones) and polysiloxane on polycarbonate. J. Appl. Polym. Sci..

[22] Zhang L., Wang Y., Liu Q., Cai X. (2016). Synergistic effects between silicon-containing flame retardant and DOPO on flame retardancy of epoxy resins. J. Therm. Anal. Calorim..

[23] Chen C.H., Chiang C.L. (2019). Preparation and characteristics of an environmentally friendly hyperbranched flame-retardant polyurethane hybrid containing nitrogen, phosphorus, and silicon. Polymers.

[24] Zhang H., Lu J., Yang H., Yang H., Lang J., Zhang Q. (2019). Synergistic flame-retardant mechanism of dicyclohexenyl aluminum hypophosphiteand nano-silica. Polymers.

[25] Yang S., Zhang Q.X., Hu Y.F. (2016). Synthesis of a novel flame retardant containing phosphorus, nitrogen and boron and its application in flame-retardant epoxy resin. Polym. Degrad. Stab..

[26] Zhang W., Li X., Yang R. (2012). Flame retardant mechanisms of phosphorus-containing polyhedral oligomeric silsesquioxane (DOPO-POSS) in polycarbonate composites. J. Appl. Polym. Sci..

[27] Wang R., Wang X.C. (2012). Research on the mechanism of a novel Si/P flame retardant. Adv. Mater. Res..

[28] Zhang J., Wang X., Zhang F., Horrocks A.R. (2004). Estimation of heat release rate for polymer–filler composites by cone calorimetry. Polym. Test..

[29] Wu N., Fu G., Yang Y., Xia M., Yun H., Wang Q. (2019). Fire safety enhancement of a highly efficient flame retardant poly(phenylphosphoryl phenylenediamine) in biodegradable poly(lactic acid). J. Hazard. Mater..

[30] Xu D., Yu K., Qian K. (2018). Thermal degradation study of rigid polyurethane foams containing tris(1-chloro-2-propyl) phosphate and modified aramid fiber. Polym. Test..

[31] Rao W.H., Zhu Z.M., Wang S.X., Wang T., Tan Y., Liao W., Zhao H.B., Wang Y.Z. (2018). A reactive phosphorus-containing polyol incorporated into flexible polyurethane foam: Self-extinguishing behavior and mechanism. Polym. Degrad. Stab..

[32] Sadezky A., Muckenhuber H., Grothe H., Niessner R., Pöschl U. (2005). Raman microspectroscopy of soot and related carbonaceous materials: Spectral analysis and structural information. Carbon.

[33] Zhao H.B., Liu B.W., Wang X.L., Chen L., Wang X.L., Wang Y.Z. (2014). A flame-retardant-free and thermo-cross-linkable copolyester: Flame-retardant and anti-dripping mode of action. Polymer.

